# Grain Boundary Character Dependence on Nucleation of Discontinuous Precipitates in Cu-Ti Alloys

**DOI:** 10.3390/ma10040415

**Published:** 2017-04-15

**Authors:** Satoshi Semboshi, Mitsutaka Sato, Yasuyuki Kaneno, Akihiro Iwase, Takayuki Takasugi

**Affiliations:** 1 Institute for Materials Research, Tohoku University, Katahira 2-1-1, Aoba-ku, Sendai 980-8577, Japan; m-sato@imr.tohoku.ac.jp; 2 Department of Materials Science, Osaka Prefecture University, Gakuen-cho 1-1, Naka-ku, Sakai 599-8531, Japan; kaneno@mtr.osakafu-u.ac.jp (Y.K.); iwase@mtr.osakafu-u.ac.jp (A.I.); takasugi@mtr.osakafu-u.ac.jp (T.T.)

**Keywords:** Cu alloy, aging, grain boundary, discontinuous precipitation, electron backscattering diffraction, misorientation, Σ value

## Abstract

The dependence of the grain boundary character distribution for a Cu-4 at. % Ti polycrystal alloy (average grain size: 100 µm) on the nucleation of cellular discontinuous precipitates was systematically investigated. In an alloy over-aged at 723 K, cellular discontinuous precipitates consisted of a terminal Cu solid solution and a stable β-Cu_4_Ti lamellae nucleated at grain boundaries. Electron backscatter diffraction analysis revealed that the discontinuous precipitation reaction preferentially occurred at random grain boundaries with a Σ value of more than 21 according to the coincidence site lattice theory. On the other hand, few cellular discontinuous precipitates nucleated at low-angle and low-Σ boundaries, particularly twin (Σ 3) boundaries. These findings suggest that the nucleation of discontinuous precipitates is closely correlated with grain boundary character and structure, and hence energy and/or diffusibility. It should therefore be possible to suppress the discontinuous precipitation reaction through control of the alloy’s grain boundary energy, by means of texture control and third elemental addition.

## 1. Introduction

Age-hardened Cu-Ti alloys have attracted interest for their application in miniature electrical components, such as connectors and lead frames, because of their excellent mechanical properties and electrical conductivity [1,2,3,4,5,6]. Cu-Ti alloys with Ti contents of approximately 3−5 at. % are commercially processed by a solid-solution treatment at a temperature of 1123 K, quenching with water, and subsequent aging at temperatures between 673 and 773 K. In the early stages of aging, the supersaturated solid solution of the fcc phase of copper begins to catastrophically decompose into Ti-rich and Ti-lean disordered fcc phases [1,7,8,9]. Later on, the Ti-rich region becomes ordered and continuously transforms into fine, needle-shaped metastable precipitates, often denoted as β′-Cu_4_Ti (tetragonal structure, proto-type: Ni_4_Mo, space group: *I*4/m), in the parent matrix phase [1,9,10,11,12,13,14,15,16]. During prolonged aging, coarse and two-phase cellular discontinuous precipitates (DPs) composed of lamellae of solute-depleted terminal Cu and stable β-Cu_4_Ti (orthorhombic structure, Au_4_Zr, *P*nma) phases nucleate and grow at the grain boundaries (GBs) [12,13,14,15,16,17,18,19].

A fine dispersion of continuous precipitates of metastable β′-Cu_4_Ti contributes to effective precipitation hardening. However, the development of coarse DPs containing lamellar-structured stable β-Cu_4_Ti is unfavorable, because it is accompanied by consumption of the favorable fine continuous precipitates. Such coarsened DPs eventually lead to serious deterioration of the mechanical and physical properties of the alloys, as seen in various commercial alloys based on Al, Cu, Co, Fe, Ni, and Pb [20,21,22,23,24,25,26]. Many investigations on this phenomenon have been previously conducted, and the nucleation and growth of DPs has also been examined [27,28,29,30]. Since DPs are generated at the GBs, the nucleation of DPs must be influenced by their character or energy. It is empirically expected that no discontinuous precipitation would occur (a) in single crystals, (b) at sub-boundaries and low-angle boundaries, or (c) at twin boundaries [31]. With regard to (a), Semboshi et al. showed that continuous precipitation took place during isothermal aging of a Cu-4 at. % Ti alloy, while no cellular precipitation was observed [32]. However, there are few systematic reports that demonstrate the feasibility of (b) and (c), at least for Cu-Ti systems. As for other systems, it was reported that the cellular reaction in a Pb-Sn bicrystal was replaced by the formation of primary side plates when the misorientation angle was slightly reduced from 15° to 13°, and from 13° to 7° for low-angle GBs [33,34]. Hornbogen et al. reported that coherent twin boundaries typically do not act as nucleation sites for cellular reactions, unless the alloy is deformed [21]. Monzen et al. reported that DPs in Cu-Be bicrystal alloys tend to nucleate at GBs structured by specific high-energy habit planes [35].

In this study, the influence of the GB character on the nucleation of DPs in a commercial age-hardenable Cu-Ti alloy was experimentally investigated. For this purpose, we utilized electron backscatter diffraction (EBSD) to characterize the GB structure of specimens before aging. After aging, regions previously characterized by EBSD were examined again by field-emission scanning electron microscopy (FESEM). In this manner, we could directly locate the nucleation sites of DPs at the GBs. By integrating a number of ‘same region observations’ by EBSD and FESEM on specimens both before and after nucleation of the DPs, the relationship between the DP nucleation sites and the character of the GBs was statistically determined and discussed in terms of the GB energy and diffusivity.

## 2. Materials and Methods

An alloy ingot with a nominal composition of Cu-4 at. % Ti was prepared by high-frequency induction melting under vacuum conditions, using pure copper (99.99 wt. %) and titanium (99.99 wt. %) strips as the raw materials. The ingot was heat-treated at 1173 K for 24 h for homogenization, after which it was cold-rolled to achieve a 95% reduction in thickness. Specimens measuring 5 mm in length, 3 mm in width, and 0.6 mm in thickness were cut from this plate. The specimens were encapsulated under vacuum (< 2.0 × 10^−3^ Pa) and then solution-treated at 1173 K for 1 h, followed by quenching in water.

The distribution of GBs in quenched specimens (before aging) was analyzed by FESEM (JEOL JSM-5100A, JEOL, Tokyo, Japan) equipped with an EBSD apparatus (DVC5 detector, TSL solutions, Sagamihara, Japan). For EBSD analysis, specimens were first mechanically polished and then electro-chemically polished with an aqueous solution of 40% phosphoric acid at room temperature using a voltage of 2.0 V for 30 s. After rinsing the specimens with ethanol, several indentations were made with a micro-hardness tester as markers, in order to define the same regions of each specimen before and after aging. Regions at least 4 mm^2^ in size, corresponding to more than 20 FESEM images at a magnification of 500×, were analyzed by EBSD with a step size of 1.0 μm (or 2.6 μm) per measurement using the orientation imaging microscopy (OIM) software (TSL solutions, Sagamihara, Japan) each area had to be wide enough to obtain statistical data (the total length of measured GBs was greater than 240 mm). The Brandon criterion [34], Δ*θ*_max_ = 15 deg∙Σ^−1/2^, was used to determine the GB character distribution in terms of the coincident site lattice (CSL) theory [36].

After EBSD analysis, specimens were re-encapsulated under vacuum and then aged at 723 K for 72 h. According to a previous study, these aging conditions reflect an early state of over-aging, during which DPs begin to develop [13,14]. Therefore, these aging conditions are useful to investigate the nucleation of DPs. The microstructures of the regions analyzed by EBSD were observed by FESEM. Prior to FESEM analysis, the specimens were subjected to light mechanical polishing using a 0.6 μm diamond paste to retain the indentations (used to mark the regions), and then chemically etched with an aqueous solution of 40% nitric acid at room temperature for 10 s.

## 3. Results

**Figure 1:**
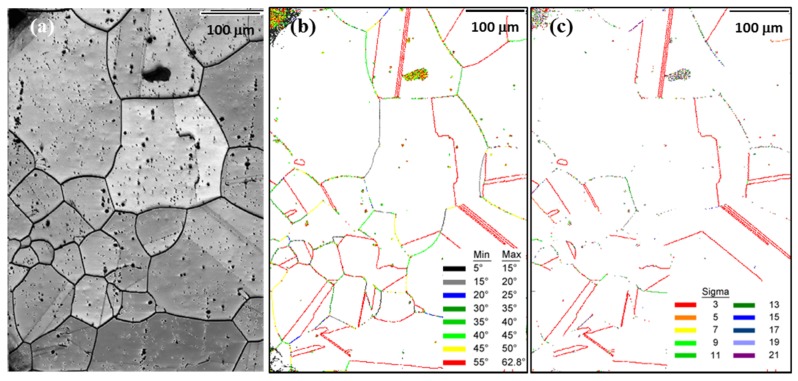
Electron backscatter diffraction (EBSD) images of the Cu-4 at. % Ti alloy specimen before aging: (**a**) image quality map; grain boundary map classified by (**b**) misorientation angle and (**c**) Σ value.

Figure 1a depicts a typical EBSD image of the Cu-4 at. % Ti specimen before aging, showing that the specimen was composed of a single phase of a supersaturated Cu solid-solution with an average grain size of approximately 100 μm. Figure 1b,c show the GB map of the specimen before aging, characterized by the misorientation angles and the Σ values, respectively. For EBSD analysis, each pair of points with a misorientation angle exceeding 5° (except for isolated pixels and noise from dirt on the surface) was considered a GB for statistical purposes. The misorientation angles presented in Figure 1b were defined by the minimum rotation angles. Thus, due to the symmetry of the cubic structure of copper, the misorientation angles had a maximum value of 62.8° [25]. In Figure 1c, high-angle GBs with Σ ≥ 3 were taken into account, and GBs with Σ > 21 were referred to as random boundaries. Note that different GB structures with the same Σ value, such as Σ 13a and Σ 13b, were not distinguished in this study. The specimen before aging had many twin boundaries, which correspond to a misorientation angle of 55–62.8° or Σ value of 3 (indicated by the red lines in Figure 1b,c).

**Figure 2:**
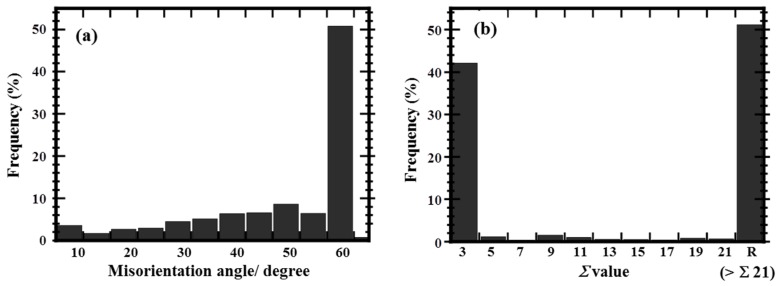
Distribution of grain boundaries for the Cu-4 at. % Ti alloy specimen before aging by (**a**) misorientation angle and (**b**) character in terms of Σ value. The misorientation angle in (**a**) is defined by the minimum rotation angle, neglecting the rotation tilt angle.

Figure 2 shows the distribution of the GBs in the Cu-4 at. % Ti polycrystalline specimens before aging as a function of the misorientation angles, as well as the Σ values, obtained from a total area of at least 4 mm^2^. The misorientation angle distribution for the specimen before aging (as shown in Figure 2a) is similar to that of heavily rolled and recrystallized pure Cu [37]. Figure 2a features a high frequency of 55–60° misorientation angles, which almost exactly correspond to twin boundaries. The latter is confirmed by the high frequency of Σ 3 twin boundaries in Figure 2b. Figure 2b also shows that most of the other high-angle GBs exhibit Σ > 21 (i.e., random GBs), although low but distinct frequencies of boundaries in the range of Σ 5–21 were detected (only 7% in total). The frequency at Σ 9 was relatively significant at 1.5%, following the Σ 3 and random GBs; this is possibly related to ‘multiple twinning’ expressed as Σ 3 + Σ 3 → Σ 9 [37,38].

**Figure 3:**
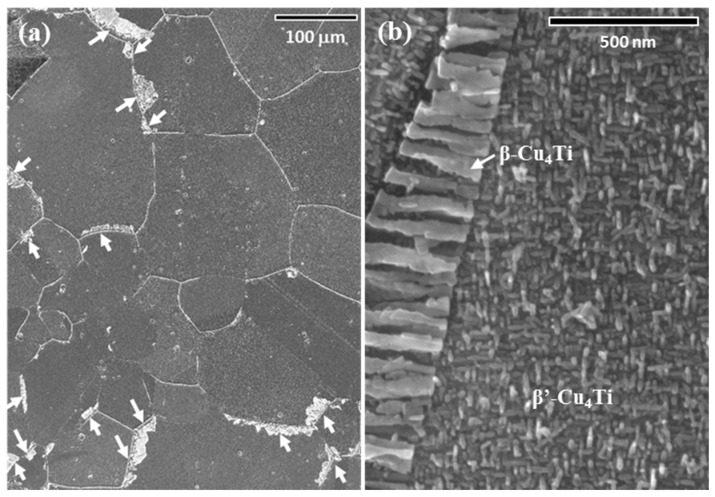
(**a**) Field-emission scanning electron microscopy (FESEM) image of the Cu-4 at. % Ti alloy aged at 723 K for 72 h, recorded at the same region as in Figure 1. The arrows indicate cellular discontinuous precipitates; (**b**) a magnified FESEM image of a grain in the same alloy as in (**a**).

Figure 3a shows an FESEM image of the specimen aged at 723 K for 72 h, at which point the DPs begin to nucleate at GBs [13,14], acquired for the same region as that shown in Figure 1a–c. These data clearly indicate no grain growth, even after aging. Cellular DPs consisting of the lamellae of solute-depleted Cu and stable β-Cu_4_Ti plates did initiate at some GBs, while continuous precipitates of fine needle-shaped β′-Cu_4_Ti emerged in the parent grains, as shown in the magnified image of Figure 3b (note that solute-depleted Cu lamellae in cellular DPs disappeared in Figure 3b due to chemical etching by the nitric acid solution). Comparing the microstructure of the specimen before aging (Figure 1b,c) with that after aging (Figure 3a) carefully, few DPs appear to have nucleated at low-angle GBs with misorientation angles of less than 15°, at twin boundaries of Σ 3, and at other boundaries with a low Σ (e.g., Σ 9 marked by green lines in Figure 1c). All of the DPs marked by white arrows in Figure 3a nucleated at random GBs of Σ > 21.

**Figure 4:**
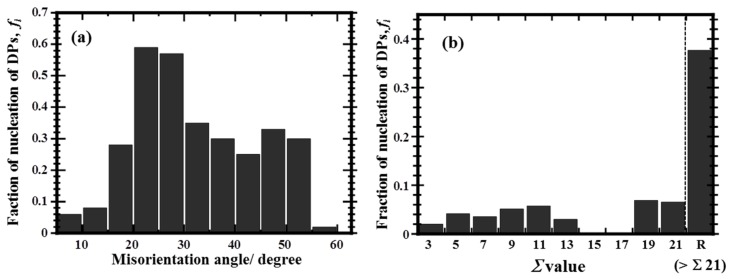
Fraction of nucleation of the discontinuous precipitates in the Cu-4 at. % Ti alloy specimen after aging at 723 K for 72 h, characterized by the grain boundary misorientation angle (**a**) and character in terms of Σ value (**b**). It should be noted that *f*_Σ__15_ and *f*_Σ__17_ are absent in (**b**) because there are no sufficient GBs belonging to Σ 15 and Σ 17 in the observed regions, as suspected from Figure 2b.

Summating the data from these observations before and after aging, we manually measured the fraction of DP nucleation, *f_i_*, as a function of the misorientation angles and Σ values, as shown in Figure 4. Here, *f_i_* is defined by the following equation:

*f_i_* = Σ*l_i_*^DP^/Σ*l_i_*^GB^ (1)

where, Σ*l_i_*^GB^ is the total length of the GB segment within a particular misorientation angle or CSL Σ value, and Σ*l_i_*^DP^ is the total length of the DPs across the GB segments. The fact that the DP may nucleate at a different GB above or below the viewing plane and grow towards the observed location was ignored, because the aging time of 72 h corresponds to an early state of DP nucleation in the alloy and the DPs are not expected to have grown significantly yet [13]. Figure 4a clearly shows that the fraction of DP nucleation was quite low at misorientation angles less than 15° for the low-angle GBs and in the range of 55–60° for the twin boundaries. The fraction of DP nucleation at the GBs exhibited peaks at misorientation angles of 20–30° and 45–55°, with a small cusp in the range of 35–45°.

Figure 4b clearly shows that the fraction of DP nucleation at the low Σ GBs was quite low, particularly at Σ 3 twin boundaries (*f*_Σ3_ = 0.02). In contrast, the fraction of DP nucleation at the random GBs, *f_R_*, exhibited a very high value of 0.38. Therefore, the nucleation of the DPs was closely related to the structure and character of the GBs in the Cu-Ti alloy specimens.

## 4. Discussion

In general, the structure and character of GBs, represented by the misorientation angles and CSL theory, can be notionally consolidated into the GB energy. In this section, the GB character-dependent nucleation of the DPs shown in Figure 4 is discussed in terms of the GB energy. First, we examine the reason for the DPs not preferentially nucleating at the low-angle GBs with misorientation angles less than 15°, as shown in Figure 4a. The structure of a low-angle GB with a misorientation angle less than 15° can be represented by an array of dislocations in a perfect lattice. Thus, the energy of low-angle GBs must increase with an increase in misorientation angle. However, it is still lower than that of high-angle GBs, except for the special low-Σ boundaries like twins [39]. Therefore, the low energy of low-angle GBs results in a lower fraction of DP nucleation.

Secondly, focusing on the high-angle GBs with misorientation angles greater than 15°, we found drops in the fraction of the DP nucleation at misorientation angles of 35–45° and 55–62.8° (Figure 4a). According to computations by Olmsted et al., the GB energy distribution in fcc materials shows cusps at misorientation angles of 35–40° and 60–62.8° [40]. Therefore, the nucleation of DPs should be suppressed at low-energy GBs.

Such a relationship between the DP nucleation and GB energy is also supported by Figure 4b. In the ‘special’ high-angle GBs, which have characteristic structures such as the Σ 3 twin boundaries and other low-Σ boundaries, the GB energy must be much smaller than that of the ‘general’ random GBs, although we should note that the energy of specific GBs depends not only on the Σ value, but also on the habit plain and rotation axis of the GB, as well as defects and strains that exist at the GB [37,40]. Thus, Figure 4b implies that the low energy of low-Σ boundaries resulted in a low fraction of the DP nucleation, while the high energy of random GBs led to a high fraction of DP nucleation.

The lowest frequency of the DP nucleation at the Σ 3 boundaries shown in Figure 4b should reflect the large cusp at misorientation angles of 55–62.8° in Figure 4a. Similarly, the low fractions of DP nucleation at Σ 5 (at a corresponding minimum misorientation angle of 36.87°), Σ 7 (38.21°), and Σ 9 (38.94°) boundaries in Figure 4b should contribute to the small cusp at 35–45° in Figure 4a, although the fraction of the actual Σ 5–9 boundaries is not significant (approximately 7%), as shown in Figure 1b.

Actually, the GB energy is closely related to the atomic free volume at the GBs; ‘specific’ GBs have a smaller atomic free volume than random GBs. A small atomic free volume at ‘specific’ GBs leads to slow GB diffusion of solute elements, which eventually causes insufficient delivery of Ti, resulting in a low frequency of DP nucleation in the Cu-Ti system. In order to validate these hypotheses, quantitative analysis of the GB diffusion coefficient of solute elements for specific and random GBs is required, which will be carried out in future studies.

As mentioned above, we have qualitatively shown that the nucleation of DPs is subject to the character and structure of GBs, and hence their energy and/or diffusion. Here, we should note again that the GB energy is not determined only by the misorientation angle and Σ value, but also by other factors such as the habit plane and rotation axis of the GB. Thus, the energy of specific GBs is not always lower than that of random GBs. Therefore, quantitative evaluation of the GB energy at the misorientation angles and Σ values in the Cu-Ti alloy system is required in order to exactly comprehend the influence of GB energy on DP nucleation probability, which will be reported in future studies.

From a practical viewpoint, it is necessary to prevent or reduce the nucleation of DPs at the GBs in Cu-Ti alloys to improve the latter’s mechanical properties. Based on the results obtained in this study, it must be possible to maximize the number of low-angle GBs and CSL or special boundaries by means of modifications of the texture pattern. It is also known that the structures and energies of GBs are affected by the segregation of solutes or impurities. Measurements of the GB energy in Ag-Au and Cu-Pb systems have previously suggested that the energies of random GBs tend to decrease with increasing GB segregation [41,42]. In a previous study on Cu-Ti alloys, in which doped boron preferentially segregated at the GBs, the nucleation of DPs at the GBs was suppressed at a later stage of aging, leading to improved hardness [43]. The concept of ‘grain boundary engineering’ therefore needs to be applied to Cu-Ti alloys in order to improve their mechanical properties.

## 5. Conclusions

In this study, the influence of GB character distribution on the nucleation probability of cellular DPs in an age-hardenable Cu-4 at. % Ti polycrystal alloy was experimentally revealed. In the early stage of over-aging at 723 K, DPs composed of a solute-depleted Cu solid solution and intermetallic β-Cu_4_Ti lamellae nucleated and grew at the GBs in the specimen. The DPs nucleated preferentially at random boundaries with Σ values greater than 21, where the fraction of DP nucleation, *f*_R_, was 0.38. On the other hand, DPs hardly formed at low-angle GBs and low-Σ boundaries, particularly twin (Σ 3) boundaries, where the fraction of DP nucleation was significantly smaller than that at random boundaries. This finding indicates that the initiation of DPs significantly depends on GB character, and hence on GB energy and/or diffusibility. It should therefore be possible to suppress the DP reaction through controlling the GB character of the alloy by means of texture control and segregation of the third element.
